# Development of Efficient and Recyclable ZnO–CuO/g–C_3_N_4_ Nanocomposite for Enhanced Adsorption of Arsenic from Wastewater

**DOI:** 10.3390/nano12223984

**Published:** 2022-11-12

**Authors:** Qudrat Ullah Khan, Nabila Begum, Zia Ur Rehman, Afaq Ullah Khan, Kamran Tahir, El Sayed M. Tag El Din, Asma A. Alothman, Mohamed A. Habila, Dahai Liu, Patrizia Bocchetta, Muhammad Sufyan Javed

**Affiliations:** 1 Greater Bay Area Institute of Precision Medicine (Guangzhou), Fudan University, Nansha District, Guangzhou 511458, China; 2 Zhongshan-Fudan Joint Innovation Center, Zhongshan 528437, China; 3 School of Medicine, Foshan University, Foshan 528000, China; 4 Department of Chemistry, The University of Haripur, Haripur 22620, Pakistan; 5 School of Chemistry and Chemical Engineering, Jiangsu University, 301 Xuefu Road, Zhenjiang 212013, China; 6 Institute of Chemical Sciences, Gomal University Dera Ismail Khan, Dera Ismail Khan 29220, Khyber Pakhtunkhwa, Pakistan; 7 Electrical Engineering Department, Faculty of Engineering & Technology, Future University in Egypt, New Cairo 11835, Egypt; 8 Department of Chemistry, College of Science, King Saud University, Riyadh 11451, Saudi Arabia; 9 Dipartimento di Ingegneria dell’Innovazione, Università del Salento, via Monteroni, 73100 Lecce, Italy; 10 School of Physical Science and Technology, Lanzhou University, Lanzhou 730000, China

**Keywords:** arsenic removal, ZnO–CuO/g–C_3_N_4_ nanocomposite, solution combustion, kinetic studies, adsorption

## Abstract

Arsenic (III) is a toxic contaminant in water bodies, especially in drinking water reservoirs, and it is a great challenge to remove it from wastewater. For the successful extraction of arsenic (III), a nanocomposite material (ZnO–CuO/g–C_3_N_4_) has been synthesized by using the solution method. The large surface area and plenty of hydroxyl groups on the nanocomposite surface offer an ideal platform for the adsorption of arsenic (III) from water. Specifically, the reduction process involves a transformation from arsenic (III) to arsenic (V), which is favorable for the attachment to the –OH group. The modified surface and purity of the nanocomposite were characterized by SEM, EDX, XRD, FT–IR, HRTEM, and BET models. Furthermore, the impact of various aspects (temperatures, pH of the medium, the concentration of adsorbing materials) on adsorption capacity has been studied. The prepared sample displays the maximum adsorption capacity of arsenic (III) to be 98% at pH ~ 3 of the medium. Notably, the adsorption mechanism of arsenic species on the surface of ZnO–CuO/g–C_3_N_4_ nanocomposite at different pH values was explained by surface complexation and structural variations. Moreover, the recycling experiment and reusability of the adsorbent indicate that a synthesized nanocomposite has much better adsorption efficiency than other adsorbents. It is concluded that the ZnO–CuO/g–C_3_N_4_ nanocomposite can be a potential candidate for the enhanced removal of arsenic from water reservoirs.

## 1. Introduction

Arsenic (III) is the most toxic and portable contaminant in nature, creating numerous environmental pollution problems worldwide as it can be effectively solubilized in groundwater [1,2]. Arsenic in water remains both in an organic as well as an inorganic state, whereas it mostly remains as arsenate (H_2_AsO_4_^−^) and oxyanions: arsenite (H_2_AsO_3_^−^) [3,4,5,6,7]. Several studies suggest that arsenic pollution can contribute to the development of different diseases, such as keratosis, melanosis, edema, and cancers of the skin, lungs, and bladder. Additionally, it has been implicated in contributing to the enlargement of the liver, kidneys, and spleen [8,9,10,11]. Consequently, the World Health Organization (WHO) has suggested that safe potable drinking water should have concentrations of arsenic no greater than 10 μg L^−1^ [12,13]. However, concentrations of arsenic in polluted water have reached levels of 100–300 μg L^−1^, which is 10–30 times higher than the maximum level recommended by WHO. This has the potential to cause severe damage to people’s health [8,14], which is 10–30 times higher than the safety limitation and will seriously damage people’s health. As a result, removing arsenic from contaminated water is a critical factor.

Up to now, different methods have been tested for the filtration of arsenic, such as reverse osmosis, adsorption, ultrafiltration, chemical precipitation, ion exchange, etc. [15,16,17,18]. Among said protocols, adsorption has many desired options for As filtration because of its effortlessness and huge efficacy [19]. However, sometimes, this protocol can be costly due to the preservation of adsorbent media. Nanotechnology solved these problems to a greater extent by providing cheap nano adsorbents with large surface areas and more specificity. Extensive efforts have been made for the adsorption of As on metal oxide nanoparticles, i.e., MgO [20], Fe_3_O_4_ [21], α-Fe_2_O_3_ [22], CeO_2_ [23], aluminium oxides [24], etc. Moreover, it has been acknowledged that the good adsorption ability of adsorbents is due to desirable active sites and large surface areas. For instance, the main limitations associated with these oxides are their agglomeration during the reaction and lesser stability, affecting their efficacy to a greater extent [25]. Cao et al. prepared a CuO nanomaterial that was applied for As adsorption but, unfortunately, they obtained a low level of As adsorption, i.e., 5.7 mg/g [26]. Therefore, it is the need of the present and the future to introduce such nanomaterials which have high efficiency and are more economical for the disintegration of arsenic. At present, metal oxides (CuO, TiO_2_, NiO, ZnO, etc.) and their combination with effective support (MCM–41, g–C_3_N_4,_ etc.) have attracted great attention from researchers in water remediation purposes because of their easy preparations, huge surface areas, and well-organized porous structures [27]. These nanomaterials have special characteristics such as high reactivity, large surface areas, regeneration capabilities, and high selectivities, which are very important for environmental remediation [28]. Various nanoparticle-based materials, for example, zinc oxide, manganese oxide, ferric oxides, and titanium oxide, are listed as effective nano adsorbents that perform well as compared to other adsorbents used for the arsenic elimination from drinking water [29,30,31,32,33]. From this perspective, high reactivity, vast surface area, and a greater number of active sites could help remove contaminants from wastewater effectively. However, their toxicity despite affordable prices, including environmental concerns, remain major problems [34,35]. Additionally, excellent adsorption efficiency and reusing the nano adsorbent are other basic requirements for efficient and selective nanomaterials that can make them an efficient candidate for removing arsenic from wastewater. The literature survey suggests a better affinity of a different metal oxide-containing adsorbent towards the elimination of arsenic due to its better selectivity and high adsorption capability for both inorganic states of arsenic As(V) and As(III) from aqueous solutions [36,37]. On the other hand, the drawback related to these nano adsorbents is accumulation owing to its low energy barrier, which greatly decreases mobility, availability, and transfers to the polluted site for in situ adsorptions. Therefore, this shortcoming can be overcome by the impregnation method, surface coating, or doping on the surface of these nano adsorbents, which could be a superior choice for the elimination of arsenic and its derivatives from polluted water. Recently, carbon nitride (g–N_3_C_4_) and its composite with metal oxides have shown better performance because of their chemical, photophysical, and catalytic properties, simple synthesis, and high stability under harsh conditions for a variety of applications, especially with photo-/electro-catalysis, sensors, and bioimaging, etc. [38,39,40,41]. Therefore, we planned to design a metal-coated nanocomposite with g–C_3_N_4_ for As removal from waste water resources.

We have prepared ZnO–CuO/g–C_3_N_4_ nanocomposites in the present contribution. A new precipitation protocol was used for the synthesis of the said nanomaterial. The synthesized nanosheets were applied as an adsorbent for As adsorption. ZnO and CuO were individually synthesized by the same protocol and used for As adsorption. Results showed that ZnO–CuO/g–C_3_N_4_ nanocomposites have several times higher As removal efficiency than their individual counterparts. The impacts of various parameters (pH, temperature, material concentration) on the efficiency of ZnO–CuO/g–C_3_N_4_ nanocomposites were also studied. Kinetics studies and adsorption isotherm models were investigated to best explain the adsorption. Finally, the detailed mechanism of arsenic adsorption was also studied.

## 2. Experimental Section

### 2.1. Synthesis of ZnO–CuO Heterostructure

Initially, the ZnO–CuO heterostructure was synthesized through a solution combustion method. Initially, 20 mL of Cu(NO_3_)2.3H_2_O at a 0.1 M concentration was mixed in a beaker with 100 mL of 0.1 M Zn(NO_3_)2.6H_2_O and stirred for up to 30 min at an ambient temperature. After that, 10 mL of 2 M sucrose solution was added as fuel into the beaker and heated at 250 °C. Finally, the hot mixture was burnt after dehydration with a flame to obtain a delicate CuO–ZnO composite powder.

### 2.2. Synthesis of g–C_3_N_4_ and ZnO–CuO/g–C_3_N_4_ Composite

Graphitic resembling C_3_N_4_ was manufactured via thermal polymerization from melamine. Typically, to obtain a yellowish powder of g–C_3_N_4_, a particular amount of melamine was placed in an alumina crucible and annealed at 550 °C for roughly 2 h in an air atmosphere. In a beaker, 1 g of g–C_3_N_4_ and 0.2 g ZnO–CuO was mixed in 70 mL water and stirred for 3 h. After 3 h, the sample was filtered and dried at 80 °C for 12 h to obtain the new material ZnO–CuO/g–C_3_N_4_.

### 2.3. Batch Adsorption Experiments

We used sodium arsenite as a primary arsenic source to test the synthesized composite adsorption characteristics in the trials. The influence of interfering ions (nitrate, phosphate, carbonate, chloride, and sulfate), pH, temperature, and time interval on the adsorption amount was determined through a batch experiment. At the same time, the kinetic reactions were additionally investigated. Initially, the pH of the reaction mixture was balanced from around 2–10, utilizing HCl (0.1 M) and NaOH (0.1 M) solutions, whereas 50 µg/L, 100 µg/L, and 150, 200 µg/L concentrations of the sodium arsenite solutions were used. Following the state of the consolidation of meddling ions with 50 µg/L, 100 µg/L, and 150 µg/L, the impact of prepared contents on the penetration of sodium arsenite with a combination of 100 µg/L was determined. Likewise, to investigate the absorption dynamism, 50 µg/L, 100 µg/L, and 150 µg/L of sodium arsenite-concentrated chemicals were checked by fixing different sampling time durations at temperatures ranging from 20 to 80 °C. The absorption equilibrium record of sodium arsenite at a starting concentration of about 20 µg/L to 200 µg/L was examined through ZnO–CuO/g–C_3_N_4_ at various temperatures (20 to 80 °C). The adsorbent was cleaned thoroughly with 50 mL of 0.1 M NaOH solutions, washed many times with distilled water, and dried up in an oven at 60 °C for three hours to recover the synthesized adsorbent. Finally, the removal rate R(%) and adsorption quantity (qe (µg/L) of arsenic through the adsorbent was determined as follows:

q_e_ = (C_0_ − C_e_)V/m (1)

%R = (1 − C_e_/C_0_) × 100 (2)

Here, C_e_ (µg/L) and C_0_ (µg/L) are the equilibrium concentration and the liquid phase initial of arsenic, m is the weight of the adsorbent utilized, and V is the volume of the liquid solution. The data obtained were processed for standard deviation and ANOVA in Excel to analyze the arsenic adsorption.

## 3. Results and Discussions

### 3.1. XRD and FT-IR Analysis

**Figure 1:**
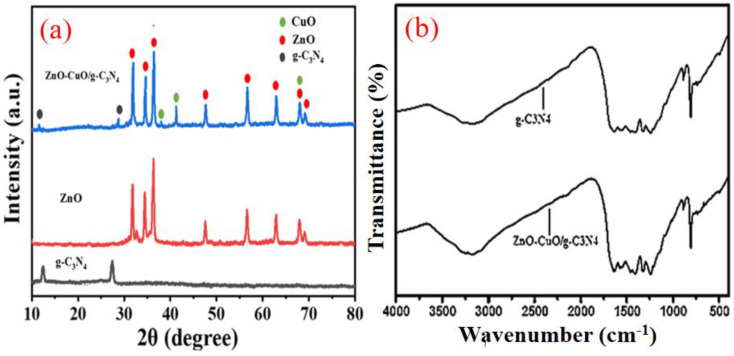
(**a**) XRD patterns of g–C_3_N_4_, ZnO and ZnO–CuO/g–C_3_N_4_ samples, (**b**) FTIR spectra of the g–C_3_N_4_ and prepared composite ZnO–CuO/g–C_3_N_4_.

Figure 1a shows the X-ray diffraction (XRD) pattern of obtained samples. From these patterns, it can be seen that the peaks situated at 2θ = 31.82°, 34.43°, 36.22°, 47.61°, 56.61°, 63.01°, and 67.98° resemble the (100), (002), (101), (102), (110), (103), and (112) planes which can be readily indexed to hexagonal wurtzite structure of ZnO (JCPDS 36-1451), respectively [42]. On the other hand, the peaks at 13.3° (100) and 27.4° (002) are ascribed to the crystal structure of g–C_3_N_4_. Consequently, the XRD spectrum of the ZnO–CuO/g–C_3_N_4_ results incorporated all the common peaks of g–C_3_N_4_, ZnO, and CuO. Additionally, CuO shows some noticeable peaks at 35.7°, 38.6°, and 67.5°, corresponding to the (002), (200), and (311), in accordance with (JCPDS card no. 89-5899). Furthermore, the peak strength intensity of the typical g–C_3_N_4_ was continuously increased with an increase in the quantity of g–C_3_N_4_, indicating a decrease in the intensity of ZnO and CuO peaks, respectively. For the ZnO–CuO/g–C_3_N_4_ sample, the XRD pattern exhibited no basic pick for g–C_3_N_4_, which can be credited to the low substance of the g–C_3_N_4_ in the composite. This result provides more explicit evidence that no extra peaks were seen in all the patterns, which indicated the high purity of the synthesized materials.

For further analysis of a composite’s surface functional groups and functional elements, we performed FT–IR spectroscopy. Figure 1b shows the FT–IR spectra of g–C_3_N_4_ and synthesized ZnO–CuO/g–C_3_N_4_ composite. The upper-level peak at around 810 cm^−1^ in the g–C_3_N_4_ test is associated with the twisting vibration attributes of heptazine moiety. At the same time, the peak observed at 3200 cm^−1^ reveals the O–H stretching vibration, suggesting the presence of moisture particles in CuO and ZnO materials. The broad group about 1200–1700 cm^−1^ in the g–C_3_N_4_ test is concerned with the ordinary stretching mode of C–N heterocycles, while another wideband about 3200 cm^−1^ in a similar case is associated with the extending vibration mode of the amine group [43]. In a composite ZnO–CuO/g–C_3_N_4_, the broad double peaks at 1631 and 1563 cm^−1^ are associated with C–N stretching vibration modes, whereas the peaks at 1251, 1323 and 1420 cm^−1^ are assigned to the aromatic C–N stretching. The broad peak around 3000 to 3500 cm^−1^ in the sample is associated with the adsorbed H_2_O and N–H vibrations of the amine groups [44].

### 3.2. HR-TEM Analysis

**Figure 2:**
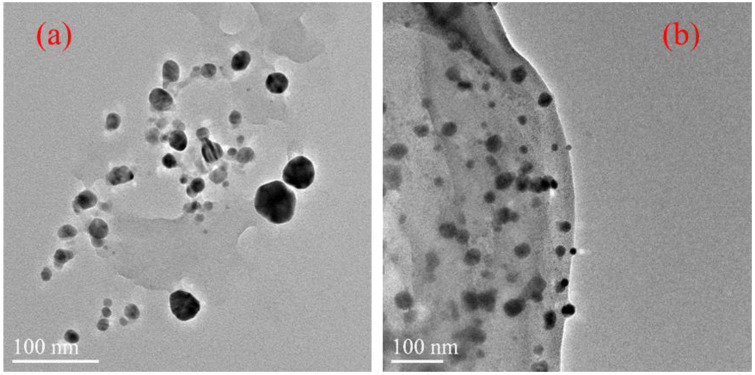
In HRTEM figure, (**a**) represents CuO–ZnO whereas (**b**) represents ZnO–CuO/g–C_3_N_4_ composites.

The structure of the as-prepared samples was observed by HRTEM, as depicted in Figure 2. The HRTEM examination of ZnO–CuO shows that ZnO and CuO have sphere morphology, control size, and are highly dispersed in nature, which consistently aligns with the XRD results, as shown in Figure 2a. As per Figure 2b, the HRTEM picture of ZnO–CuO/g–C_3_N_4_ indicates its covered platelet-like morphology and plain paper-fold diluent sheet, which is identical to the design of the nanosheets of graphene [45]. The ZnO and CuO particles are dispersed uniformly with a small size on the face of g–C_3_N_4_ nanosheets. It confirms the successful synthesis of ZnO–CuO/g–C_3_N_4_ heterostructure composite [46].

### 3.3. SEM and EDX Analysis

**Figure 3:**
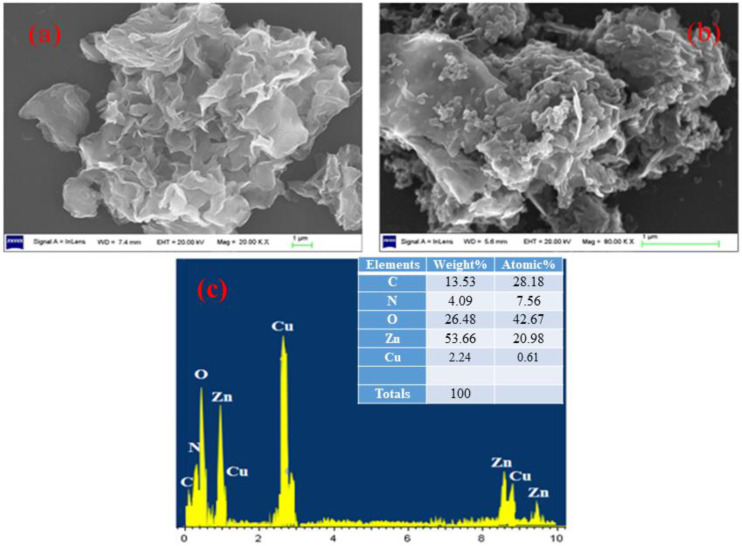
SEM images of (**a**) of g–C_3_N_4_, (**b**) ZnO–CuO/g–C_3_N_4_, and (**c**) EDS analysis of ZnO–CuO/g–C_3_N_4_ composite.

To test the fluctuation in material morphology in response to CuO and ZnO charging, EDX and SEM studies were applied to perceive the external structure and chemical configuration of ZnO–CuO/g–C_3_N_4_. Figure 3a represents the structure of the g–C_3_N_4_ as having sheet-like morphology with a large size and a higher degree of aggregation, whereas in the case of the ZnO–CuO/g–C_3_N_4_ composite, the tiny nanoparticles of ZnO and CuO are dispersed on the surface of the thin sheets to overcome the aggregation and enhance the surface area of the composite [47], as shown in Figure 3b. To validate the purity of the synthesized composite further, the EDX study was conducted. Figure 3c depicts that the existence of Zn, Cu, C, O, and N in the nanocomposite ensures the purity of the synthesized composite.

### 3.4. Nitrogen Adsorption-Desorption Study

**Figure 4:**
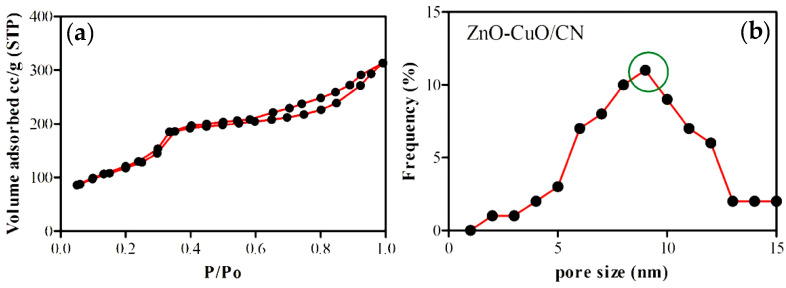
(**a**) N_2_ adsorption-desorption isotherm curves of the synthesized ZnO–CuO/g–C_3_N_4_ and (**b**) their pore size distribution.

Figure 4 depicts the N_2_ adsorption-desorption isotherms and the Barrett-Joyner-Halenda pore-size dispersion bends of ZnO–CuO/g–C_3_N_4_ nanosheets. The Brunauer-Emmett-Teller (BET) explicit surface territory for CuO nanosheets ZnO–CuO/g–C_3_N_4_ is 268 m^2^ g^−1^. The pore-size dispersions are at a maximum for the nanosheets and are about 4 nm each. The highly specific area of ZnO–CuO/g–C_3_N_4_ nanosheets might be because of the porous structure of nanosheets, which is good for electrochemical applications. ZnO–CuO/g–C_3_N_4_ nanosheets yield a large, exposed surface area designed for the adsorption of particles and charge transfer reactions. Figure 4B illustrates the pore size distribution of the synthesized ZnO–CuO/g–C_3_N_4_ nanomaterial. It is clear from the figure that most of the pores have a diameter between 8–12 nm, which corresponds to the mesoporous structure of the material [48].

#### XPS Examination

**Figure 5:**
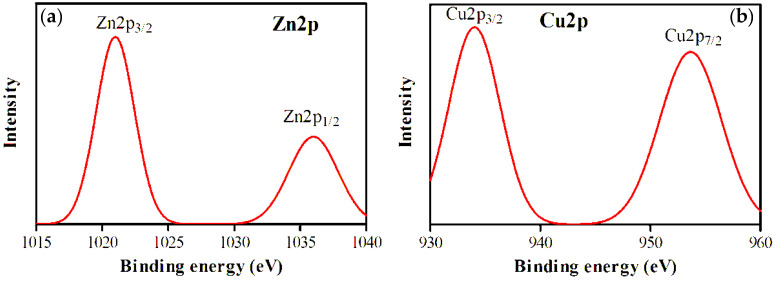
XPS analysis of synthesized nanocomposites, (**a**) Zn-2p and (**b**) Cu-2p.

XPS was applied to study the type of bonding and % age weight of each element present in the sample. Figure 5a,b represent the XPS analysis of ZnO and CuO, respectively. The result illustrated (Figure 5a) that two well-examined peaks are present at 1021.6 eV and 1034.3 eV, corresponding to Zn 2p_3/2_ and 2p_1/2,_ respectively. The two very intense peaks originating at 933.7 eV and 953.8 eV are suggested for Cu 2p_3/2_ and 2p_1/2_, respectively (Figure 5b). Similarly, the XPS also confirmed the % weight of Zn and Cu, which are 26.7% and 31.22%, respectively.

### 3.5. Batch Adsorption Experiment

**Figure 6:**
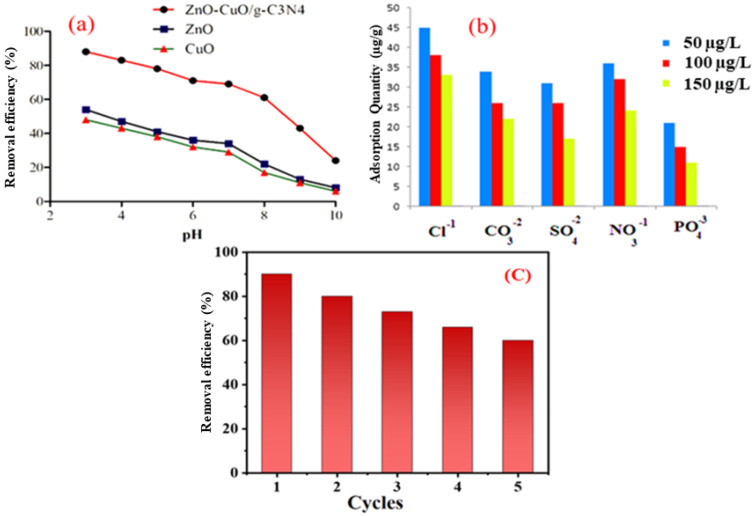
(**a**) Effect of pH (**b**) interfering ions on the removal of As(III) by ZnO–CuO/g–C_3_N_4_ composite, (**c**) removal rate (%) of As(III) by ZnO–CuO/g–C_3_N_4_ nanomaterial composites in five recycling steps.

The water pH may impact the expulsion of arsenic by adsorbent material. To enhance the evacuation effectiveness of synthesized compounds in an actual application, the adsorption of three different samples of sodium arsenite (150, 100, 50 µg/L) was investigated under a range of pH, as it appears in Figure 6a. The shifting pattern of the curves revealed that acidic environments are beneficial for the removal of arsenic by ZnO–CuO/g–C_3_N_4_ material. In contrast, adsorption decreases at high pH levels, which is aligned with the prior studies [49]. It is additionally crucial that the expected pH value of the sodium arsenite solution is around 8; when the underlying pH is adapted to about 2.8, the adsorption capacity reports a total increasing tendency with a bit of increment adequacy. This is because, under corrosive conditions, an enormous quantity of H^+^ ions can make adsorptive surfaces protonated and positively charged. Thus, it is easier to enhance the elimination of arsenic radicals by electrostatic forces. Although, when the pH ranged from basic up to 10, the adsorption capacity declined significantly. After being prewetted with water, the −OH ions may acquire the adsorption site with arsenite. The hydroxides of metal ions are created after adoption, which would stop the response from carrying on. In this way, examining pH supports choosing the ideal adsorbent in a reasonable application.

Taking into consideration the complicated chemical characteristics of normal water, the impacts of a few ordinary interfering ions (PO_4_^3−^, NO_3_^−^, SO_4_^2−^, CO_3_^2−^ and Cl^−^) with three initial dilutions (50, 100, 150 µg/L) on As(III) elimination by ZnO–CuO/g–C_3_N_4_ were tested, as can be observed in Figure 6b. Undoubtedly, the snooping of the five common ions decreased the adsorption limit, and the higher the number of active ions, the more significant the influence on adsorption capacity. Chloride ions have a minimal impact on the adsorption amount due to the additional framing of Cl^−^ ions by spheric complexes with a heterostructure composite. By correlation, the nearness of sulfate and carbonate affects the adsorption amount, particularly in the description that sulfate ions and carbonate ions convey progressively extra negative charges and occupy additional adsorption sites, which eventually decrease the removal proficiency of arsenic. Nitrate conveys a smaller amount of negative charge; therefore, its impacts on arsenic adsorption are firmly trailed by that of chloride ions. However, with high concentrations of conjunction anion quantities, seriously good adsorption develops stronger. Moreover, it is evident from the graph in Figure 6b that phosphate has the potential to interfere, and its existence seriously decreases the adsorption amount. In this examination, the concentration of contending anions was set much higher than those experienced in natural water. Consequently, even though confronting anions concentrations used in these experiments are unusually higher, they still have the adsorption ability for arsenic exclusion. Recyclability is one of the significant records to assess the application of adsorbents. Subsequently, the following study showed a sodium arsenite solution with a starting concentration of 50 µg/L as the trial object, and the adsorbent was 10 mg of ZnO–CuO/g–C_3_N_4_ that adsorbed in 24 h.

On the other hand, a 0.1 M NaOH solution was used for the desorption of adsorbed materials, and the recycling experiments were repeated five times for the arsenic removal rate, as shown in Figure 6c, examining the reusability and stability of the prepared materials. Latterly, it tends to be the case that the exclusion percentage of the first cycle is as elevated as 90%. Still, the proficiency of the second exclusion cycle is reduced to about 80% because of several adsorption sites being filled and not desorbed. Furthermore, the elimination rates of the previous three cycles were not significantly different, showing that physio-adsorption may be dominant for the time being. The fifth cycle’s As(IV) elimination rate can still reach above 60%, demonstrating that the synthesized materials, ZnO–CuO/g–C_3_N_4_, have excellent stability and recyclability and are projected to be functional in water purification.

### 3.6. Effect of Physical Parameters on the Adsorption of Arsenic

**Figure 7:**
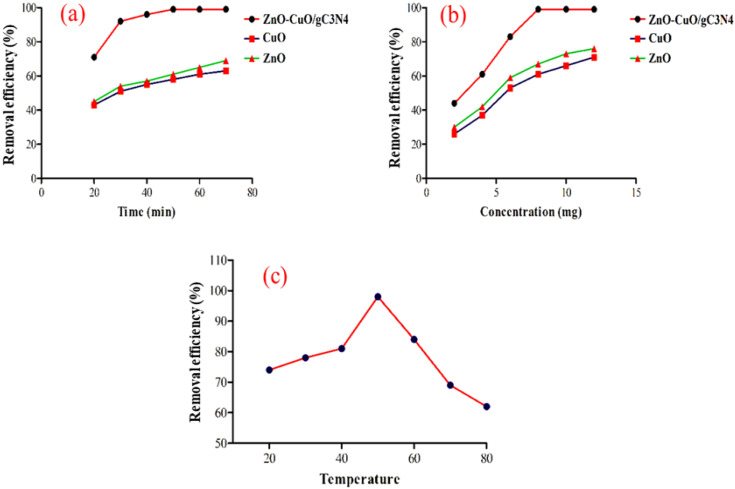
(**a**) Effect of time, (**b**) concentration of nanomaterial, (**c**) temperature on the adsorption of arsenic.

The effect of various physical parameters such as time, the concentration of nanomaterial, and temperature on the adsorption of arsenic was also examined at multiple time intervals in the presence of ZnO, CuO, and ZnO–CuO/g–C_3_N_4_ nanocomposites (Figure 7a). It was noticed that the adsorption capability of said nanomaterial improved with a rise in time by fixing the temperature at 50, pH at 4, and concentration of nanomaterial at 7 mg. 98% adsorption of As, which was achieved after 70 min of stirring.

The arsenic adsorption capability of the as-synthesized ZnO, CuO, and ZnO–CuO/g–C_3_N_4_ nanocomposites were assessed at various nanomaterial amounts, i.e., 2 to 12 mg, as depicted in (Figure 7b). The result showed that As adsorption is directly related to the amount of nanomaterial used and a percent decrease (50–98%) of arsenic was observed from 2–7 mg of the nanomaterial used. Further, an increase in the amount of ZnO–CuO/g–C_3_N_4_ nanocomposites at specific optimized conditions does not affect the adsorption of arsenic because almost all arsenic is adsorbed at lower concentrations of the said nanomaterial. Less than 70% As was adsorbed by individual ZnO and CuO even at a concentration of 12 mg. Temperature also significantly affects As adsorption. In Figure 7c, the result showed that with an increase in temperature from 20 to 50 °C, the adsorption capability of the synthesized nanomaterials increases. However, beyond 50 °C, the adsorption capability of nanomaterials decreases sharply, which may be due to chemisorption fruitfully occurring at 50 °C. As adsorption also occurs at a lower temperature, physisorption also occurs side by side with chemisorption. All these results are well proven by Freundlich and Langmuir isotherms.

### 3.7. Adsorption Isotherms

**Figure 8:**
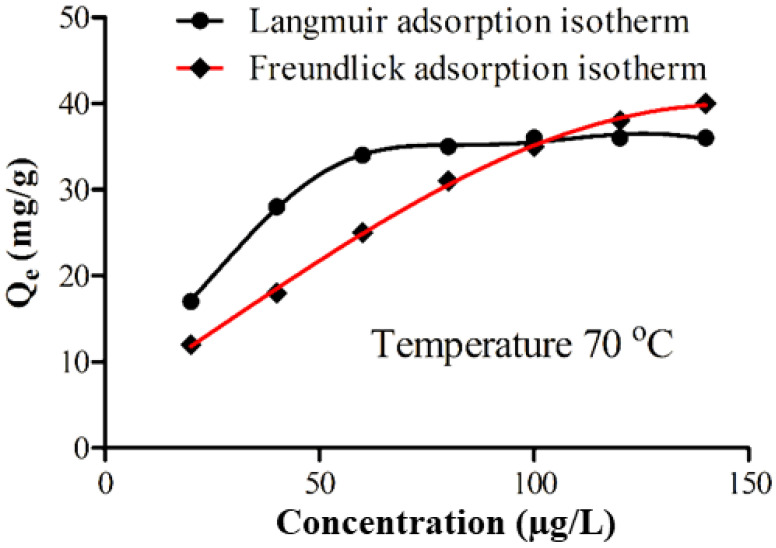
Freundlich and Langmuir isotherms of As(III) adsorption on ZnO–CuO/g–C_3_N_4_.

To study the relationship between adsorption capacity and equilibrium concentration at different temperatures (20 to 80 °C), adsorption experimentation of sodium arsenite solution with starting concentration of 20–150 µg/L was performed for one day with an equimolar mass of adsorbent. On this premise, the popular Freundlich and Langmuir adsorption isotherm was utilized to fit the tested experimental data and to know the type of adsorption and higher arsenic adsorption ability; the results are shown in the form of the adsorption isotherm in Figure 8. Langmuir’s calculations are dependent on the following perceptions: (1) The adsorbate accumulates on the upper surface of the adsorbent in the form of a monolayer; (2) limited adsorbent adsorption capacity; (3) the adsorbed particles do not interact with one another because they have the same reactive sites. The Freundlich model depicts multilayer adsorption and displays the variability of the adsorbent surface. The associated equations of the two models can be determined from Equations (3) and (4) [50,51].

Q_e_ = Q_m_K_L_C_e_/1 + K_L_C_e_ (3)

Q_e_ = KFC1/n e (4)

Here, C_e_ (µg/L) is the equilibrium concentration of adsorbate, Q_m_ (mg/g) and Q_e_ (mg/g) is the quantity of adsorbent absorbed at an equilibrium stage of C_e_ and the theoretical highest adsorption capacity, respectively; KF (mg/g) and K_L_ (µg/L) (µg/L) 1/n is the Freundlich constant and the Langmuir constant, correspondingly; and n is the heterogeneity factor. It tends to be naturally seen from Figure 8 that every one of the three curves displays a rising trend; however, there is no eternal ascending pattern. When the curve rises to a definite level, it could be flat due to the adsorption saturation. Additionally, the temperature also has a high impact on the adsorption ability. At a similar starting concentration, a temperature change can cause a difference in equilibrium concentration, affecting adsorption capabilities, and the law states that low temperatures are more favorable for adsorption. The boundaries of the Langmuir and Freundlich models are determined by association with Equation (1). Contrasting the R^2^ of the two models, the R^2^ fit by Langmuir (0.989) was higher than the R^2^ fit by Freundlich (0.973), implying that the Langmuir model adequately exhibited the adsorption isotherms. The findings of this study revealed that arsenite was attached on the outside with confined and uniform sites, with a monolayer adsorption site. It can be naturally observed that acidic or neutral water is helpful for the adsorption of arsenic, which might be correlated with the diverse compositions of the prepared materials. Moreover, a comparison of different adsorbents with our designed ZnO–CuO/g–C_3_N_4_ adsorbent for arsenic removal is summarized in Table 1. The consequences recommended that ZnO–CuO/g–C_3_N_4_ was not just economically cheaper and ecologically safe in cases of raw ingredients, but also had a higher proficiency for arsenic removal.

### 3.8. Adsorption Kinetics

**Figure 9:**
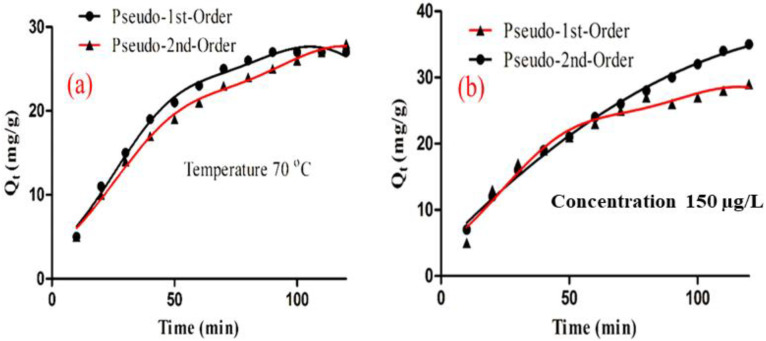
Kinetic study of As(III) removal by ZnO–CuO/g–C_3_N_4_ at (**a**) Temperature and (**b**) Concentration versus time intervals.

In adsorption kinetics, the relationship between the adsorption limit and time is investigated for various starting concentrations and temperatures of sodium arsenite solution. Moreover, Figure 9a displays the outcomes of adsorption amounts fluctuating at a certain temperature (70 °C) following the combination of a sodium arsenite solution with an underlying dilution of 150 µg/L consumed by a specific quantity of adsorptive material. Furthermore, it tends to be naturally observed that the adsorption volume expanded quickly in the initial 60 min and gradually expanded after the fixed 60 min. Still, the curve bends inclined toward the plane after 120 min. Since there were countless adsorption positions bringing adsorption from the start, arsenite particles responded to cupric oxide until the surface was fully occupied with plenty of hydroxyl functional clusters for adhesion. The place could be filled progressively with a gradual increase in adsorption quantity until all the sites were entirely occupied, leading to a saturated state. Likewise, various temperatures reported diverse adsorption capacities under similar conditions, and the standard is 70 °C, which is reliable with the above-stated results of the adsorption isotherm. Figure 9b shows the determined results for the change of adsorption quantity versus time intervals under multiple starting dilutions of 50 µg/L, 100 µg/L, and 150 µg/L at a temperature of 70 °C. At the start, the capacity of adsorption abruptly increases with time. It later slowly improves to the equilibrium state, and adsorption capability is larger for the higher concentration than for the lower concentration. It can be concluded that the capacity of adsorption increases at changing the degree of temperature and starting concentration with time. Adsorption kinetics may give valuable evidence for the entire adsorption method. For a deeper understanding of the impact of varying temperatures and to start focusing on the adsorption velocity, pseudo-first-order kinetics (Equation (5)) and 2nd-order kinetics (Equation (6)) were chosen to examine the experimental results extensively. Moreover, the kinetic equation of pseudo-first-order and pseudo-second-order is represented as follows:

ln(Qe − Qt) = lnQe − K_1_t (5)

t Qt = t Qe + 1 K_2_Q_2_ e (6)

The quantities of As(III) adsorbed by adsorbents at equilibrium and at time t, respectively, are Q_e_ and Q_t_ (mg/g), and the rate constants for the pseudo-first-order and pseudo-second-order models, respectively, are k_1_ (1/min) and k_2_ (g/(mg min).

The value of R^2^ shows that adsorption capacity was reported in a good arrangement in the presence of a pseudo-second-order dynamic model, where the connection temperature was already adjusted to 70 °C, and the starting focus of sodium arsenite was fixed at 50 µg/L and 100 µg/L. The outcome demonstrates that copper oxide is engaged with the reaction. The R^2^ achieved via pseudo-first-order and pseudo-second-order dynamics are prominently dissimilar, demonstrating that typical adsorption is also found, primarily because of the copper oxide. Furthermore, a visible decline is seen in the constant rate of the pseudo-first-order kinetics model (K_1_) and the pseudo-second-order kinetics model (K_2_) with an increase in the temperature of the reaction. This demonstrates that either chemical or physical adsorption prevails, and the required time to bring the equilibrium in adsorption could increase in response to a rise in reaction temperature. In contrast, adsorption efficiency is improved at a lower temperature. On account of altering the starting concentrations, the constant rate of the pseudo-first-order kinetic model (K_1_) is reported as 0.044 correspondingly, indicating that the concentration fluctuation has a slight impact on the typical adsorption process. The study of adsorption kinetic gives essential and valuable information to examine the process of adsorption efficiency and physicochemical responses.

### 3.9. Statistical Analysis

All the adsorption data were analyzed statistically to check the accessibility of the applied adsorption model and the efficiency of the synthesized adsorbent (ZnO–CuO/g–C_3_N_4_). A two-tailed *t*-test at a 5% significance level was used to confirm the optimum pH, and a Paired *t*-test was used to check the experiment’s success.

#### 3.9.1. Hypothesis Confirming Optimum pH of Adsorption of Arsenic (III)

Two hypotheses, null and alternate, were assumed to confirm the optimum pH by a two-tailed *t*-test at a 5% level. The maximum adsorption at various pH is given in Table 2.

Null Hypothesis = Optimum pH is equal to 3

Alternate Hypothesis = Optimum pH is not equal to 3

*T_observed_* was calculated using Equation (7):

(7) $$t_{observed}=\frac{X_{avg}-\mu }{\sigma_{s}/\surd n}$$

In Equation (8), *μ* is the optimum pH, whereas $\sigma_{s}$ is the standard deviation, and it was calculated by using Equation (8).

(8) $$\sigma_{s}=\surd [\;\sum \left(X_{i\;}–\;X_{avg\;}{)}^{2\;}/\left(n-1\right)\right]$$

*T_observed_* was found to be 0.92 and was compared with *t_tabulated_* (2.306). As *t_observed_* is less then *t_tabulated_*_,_ the null hypothesis is accepted, and optimum pH = 3 for maximum adsorption is confirmed.

#### 3.9.2. Hypothesis to Confirm the Success of the Experiment Using ZnO–CuO/g–C_3_N_4_ as Adsorbent

The success of the experiment was confirmed by proving that the concentration of arsenic (III) changes during the adsorption process. A Paired *t*-test was applied to the data given in Table 3 to test.

Hypothesis. The two hypotheses were:

Null Hypothesis: No change in adsorbate concentration.

Alternate Hypothesis: During adsorption, adsorbate concentration changes.

(9) $$t_{calculated\;}=\frac{D_{avg}}{\sigma_{diff\;}}\;\surd n$$

(10) $$\sigma_{diff\;}=\frac{\sqrt{\left[\;\sum {\left(D_{i\;}-D_{avg}\right)}^{2\;}\right]n}}{n-1}$$

$\sigma_{diff\;}$ was calculated by Equation (5) 26.11 was found. In addition, the *t* value was calculated by Equation (4) which comes out to be 4.19, and it was compared with *t* tabulated (2.77). As *t_calculated_* is greater than the tabulation, the null hypothesis is rejected and the alternate hypothesis, arsenic (III) concentration changes during adsorption, is accepted.

### 3.10. Mechanism of As(III) Removal

**Scheme 1:**
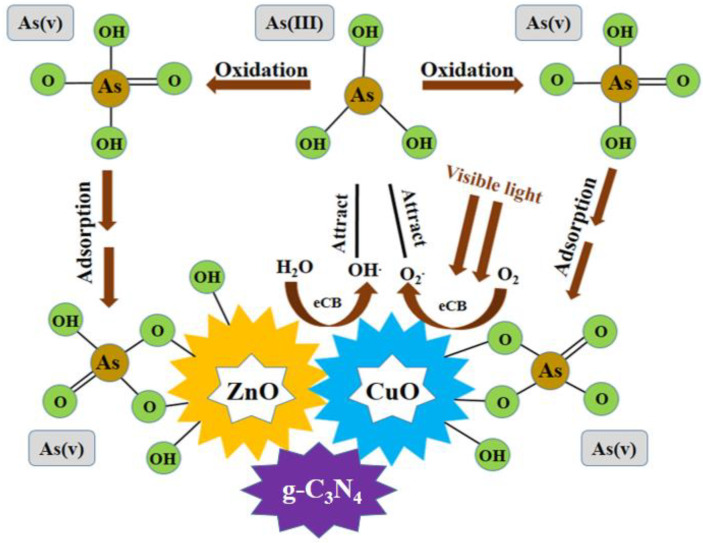
Shows the schematic diagram for the removal mechanism of arsenic.

The method of arsenic adsorption by material ZnO–CuO/g–C_3_N_4_ depends on the combined impact of redox reaction and physical interaction, and Scheme 1 shows the graphic design for the removal mechanism of arsenic. The preparation of a single CuO can result in severe accumulation, mainly decreasing the efficiency of the substance, but g–C_3_N_4_ based as the substrate not only resolved the environmental challenges affected by the single CuO and ZnO but also resolved the faults of accumulation, thus significantly enhancing the reactivity of materials and the adsorption proficiency. Moreover, the surface of g–C_3_N_4_ has plenty of –OH groups, which are beneficial for connecting copper ions. In the case of aqueous media, the progression of the hydrothermal process may result in copper ions formation, which can mostly create a CuO layer on the upper surface of g–C_3_N_4_ as per the “Seed growth method”. Therefore, the g–C_3_N_4_ is an essential support for the CuO and ZnO to attain the highest proficiency. During the reactions, when the adsorbent comes in cross-contact with the arsenite ion, CuO oxidizes the arsenic trivalent (As (III)) into the arsenic pentavalent (As (V)). In the FT–IR spectra, the peak depth for –OH and CuO decreases, and enlightening hydroxyl is used during the adsorption. Accordingly, the partly oxidized arsenate ion might come in contact with the surface and respond with a –OH substitution, or it might be that As (III) directly attaches to the –OH. Convincingly, the determination of the mechanism indicates that the prepared ZnO–CuO/g–C_3_N_4_ can extract As from both physical and chemical reactions, and the combined impact of g–C_3_N_4_, ZnO, and CuO highly enhance the proficiency of arsenic removal. Another proposed mechanism is producing reactive oxygen species (ROS) in visible light on the surface of CuO and ZnO. The ROS produced may be hydroxyl radicals or superoxides. These ROS are easily obtained in the presence of ZnO, CuO, and g–C3N4 because all of these have shallow band gaps, and the ground electrons are easily subjected to an excited state even in ordinary visible light. After producing these oxygen species, they reacted with arsenic and adsorbed on the synthesized nanomaterial surface.

## 4. Conclusions

In brief, ZnO–CuO/g–C_3_N_4_ nanomaterial was synthesized by grafting ZnO–CuO onto g–C_3_N_4_. The shape, surface area, morphology, and stability of the synthesized nanostructures were confirmed by different analytical techniques. The result showed that the synthesized nanostructure could effectively remove arsenic from the water solution. Various factors affecting the adsorption efficiency of the said nanomaterials were also explored. The adsorption of arsenic increases with a decrease in pH, nanomaterials’ concentration, and temperature (50 °C) was noted. Beyond this temperature, the adsorption efficacy of the as-synthesized nanomaterials decreased. Additionally, ZnO–CuO/g–C_3_N_4_ showed enhanced recycling activity in five consecutive cycles. The adsorption isotherm results showed that experimental data matches the Langmuir model well, suggesting that the adsorption approach is specific to a suitable site. The adsorption kinetic data exhibited that the maximum adsorption capacity of the as-synthesized composite by Langmuir and Freundlich was observed as 0.989 and 0.973 mg g^−1^, respectively. Among the pseudo-second-order kinetic model and the pseudo-first-order kinetic model, there was no discernible difference in R^2^. Consequently, our designed ZnO–CuO/g–C_3_N_4_ composites are a promising material for actual application in environmental remediation.

## Figures and Tables

**Table 1:** Comparison of the maximum arsenic adsorption capacity among the prepared ZnO–CuO/g–C_3_N_4_ nanocomposite and other adsorbents.

Adsorbents	Adsorption Capacity	References
	As (mg·g^−1^)	
Reduced graphene oxide-supported mesoporous Fe_2_O_3_/TiO_2_ nanoparticles	99.5	[52]
CuFe_2_O_4_ foam	85.4	[53]
Zirconium nanoscale carbon	110	[54]
(Layered double hydroxides/graphene oxide) nanocomposites	183.11	[55]
MnFe_2_O_4_	90	[56]
CoFe_2_O_4_	74	[56]
GO–MnFe_2_O_4_	207	[57]
Fe_3_O_4_	116.56	[58]
Ce–Fe Oxide–carbon nanotubes	30.96	[33]
Cellulose-based composites	83	[59]
Copper oxide (II) nanoparticles	88.3	[44]
ZnO–CuO/g–N_3_C_4_	97.56	Current work

**Table 2:** Maximum removal of arsenic (III) at various pH.

*n*	pH	% Removal (*X_i_*)	(*X_i_*− *X_avg_*)^2^
1	3	98	915.06
2	4	92	588.06
3	5	84	264.06
4	6	73	27.56
5	7	65	7.56
6	8	61	45.56
7	9	43	612.56
8	10	26	1743.06
		*X_avg_* = 67.75	*∑*(*X_i_* − *X_avg_*)^2^ = 4203.48

**Table 3:** Change in concentration of arsenic(III) during adsorption.

*n*	X_i_ (Arsenic Initial Concentration in ppm)	X_f_ (Arsenic Final Concentration in ppm)	*D_i_* = *X_i_* − *X_f_*	(*D_i_* − *D_avg_*)^2^
1	20	3.5	16.5	1054.95
2	40	4.7	35.3	187.14
3	60	6.8	53.2	17.80
4	80	14.7	65.3	266.34
5	100	25.4	74.6	656.38
			*D_avg_* = 48.98	∑(*D_i_* − *D_avg_*)^2^ = 2182.6
